# Deep Sequencing Reveals New Aspects of Progesterone Receptor Signaling in Breast Cancer Cells

**DOI:** 10.1371/journal.pone.0098404

**Published:** 2014-06-04

**Authors:** Anastasia Kougioumtzi, Panayiotis Tsaparas, Angeliki Magklara

**Affiliations:** 1 Department of Biological Applications and Technologies, University of Ioannina, Ioannina, Greece; 2 Department of Computer Science and Engineering, University of Ioannina, Ioannina, Greece; 3 Laboratory of Clinical Chemistry, School of Medicine, University of Ioannina, Ioannina, Greece; 4 Foundation of Research and Technology-Hellas, Institute of Molecular Biology & Biotechnology, Department of Biomedical Research, Ioannina, Greece; University of Patras, Greece

## Abstract

Despite the pleiotropic effects of the progesterone receptor in breast cancer, the molecular mechanisms in play remain largely unknown. To gain a global view of the PR-orchestrated networks, we used next-generation sequencing to determine the progestin-regulated transcriptome in T47D breast cancer cells. We identify a large number of PR target genes involved in critical cellular programs, such as regulation of transcription, apoptosis, cell motion and angiogenesis. Integration of the transcriptomic data with the PR-binding profiling of hormonally treated cells identifies numerous components of the small-GTPases signaling pathways as direct PR targets. Progestin-induced deregulation of the small GTPases may contribute to the PR's role in mammary tumorigenesis. Transcript expression analysis reveals significant expression changes of specific transcript variants in response to the extracellular hormonal stimulus. Using the *NET1* gene as an example, we show that the PR can dictate alternative promoter usage leading to the upregulation of an isoform that may play a role in metastatic breast cancer. Future studies should aim to characterize these selectively regulated variants and evaluate their clinical utility in prognosis and targeted therapy of hormonally responsive breast tumors.

## Introduction

Progesterone is a steroid hormone that plays a pivotal role in female physiology by co-coordinating diverse aspects of the reproductive system [1] and regulating mammary gland morphogenesis [2]. It exerts its actions through the two isoforms of the progesterone receptor (PR-A and PR-B), which are ligand-activated transcription factors that belong to the nuclear receptor super-family. Deregulation of progesterone signaling is implicated in the development and progression of cancer in the hormone's target tissues [3]. In breast cancer the role of PR is well documented both *in vivo* and *in vitro* [4]. Experiments in PR knock-out mice demonstrated that progestins promote mammary tumor progression and growth [2], [5], [6]. Two large clinical studies in women [7], [8] have also provided supporting evidence for a tumorigenic role of progesterone in the mammary tissue. *In vitro* studies have confirmed that progestin treatment affects important cellular programs, such as proliferation, apoptosis and differentiation [3], [9], all of which have the potential to lead to a malignant phenotype when deregulated.

To develop effective therapeutic schemes against PR signaling in breast cancer, a major requirement is the determination of the full repertoire of progestin-regulated genes in target cells. Gene expression microarray studies have been useful in characterizing transcriptional effects of progestin signaling [10], [11], [12], [13], [14], [15]. However, this approach is lacking due to high levels of noise, relatively low sensitivity and limited number of array probes suggesting that a plethora of PR-regulated genes may still remain undetected.

More than 90% of human genes can generate multiple transcript variants, which are, oftentimes, designated with tissue- or developmental- specific functional roles [16]. A growing number of studies have demonstrated the expression of cancer-associated variants participating in specific cellular programs, including apoptosis, cell growth, angiogenesis and cell motility, during tumor initiation and progression [17], [18]. Detection and characterization of such variants can improve our understanding of the molecular mechanisms in play; it can also have significant impact in the clinic, since they have emerged as a promising tool for the diagnosis and management of the disease [19], [20]. Studies using exon-specific microarrays have identified estrogen-regulated transcript variants in breast cancer cell lines [21], [22]. However, it is currently unknown to what extent progestin-regulated transcript variants contribute to the expression profile of breast cancer cells. The gene expression microarrays studies described above could not discriminate between variants and the reports of up- or down-regulation of mRNA expression levels are confounded by the effects of mixtures of these transcripts [19].

To address the above issues, we employed paired-end, next-generation sequencing (NGS) to interrogate the transcriptome of vehicle- and progestin- treated T47D breast cancer cells in an unbiased way. We identified hundreds of PR regulated genes that participate in important cellular processes, including apoptosis and transcription as reported before [14], but also angiogenesis and cell migration. More importantly, we identified a novel group of PR targets that are involved in small-GTPases signaling. By employing ChIP-seq experiments for the PR, we showed that many of these genes were under direct progestin transcriptional regulation via the receptor's binding in their promoters or distal enhancer elements. Small-GTPases signaling pathways are widely implicated in normal physiology and disease [23]. According to our data some of them may be subject to PR-regulation and may be mediating the receptor's effects in breast cancer cells. On the transcript level we find that the receptor can dictate alternative promoter use decisions leading to significant expression changes of specific transcript variants as a response to the external hormonal stimulus. Transcript variants often encode for unique protein isoforms with different, even antagonizing, functions [24]; consequently, expression data on the transcript level are necessary to paint an accurate picture of the PR-regulated proteome.

In overall, our findings provide new insights into the molecular mechanisms of PR signaling and the progestin-regulated transcriptome of breast cancer cells.

## Materials and Methods

### Cell culture and Reagents

T47D cells were purchased from ATCC and were grown in RPMI supplemented with 10% fetal calf serum and glutamine (Life Technologies) at 37°C under 5% CO_2._ Before hormonal treatment (10 nM R5020) cells were plated in RPMI/charcoal-stripped fetal bovine serum either 24 hours (RNA isolation) or 3 days (ChIP assays) before harvesting. The α-PR antibody (catalog no. sc-7208) was purchased from Santa Cruz Biotechnology, Inc. (Santa Cruz, CA), the α-PolII was from Covance (catalog no. MMS-126R) and the antibody against mono/di/trimethyl-Histone H3 (Lys4) was from Millipore (clone AW304 catalog no 04-791).

### Reverse Transcription qPCR

Total RNA was extracted from cells using the Trizol reagent (Life Technologies), and 2.5 µg of RNA were reverse transcribed using the RevertAid First-Strand cDNA Synthesis System (ThermoCcientific) according to the manufacturers' instructions. cDNA was amplified by quantitative PCR (qPCR) using the SYBR FAST Universal 2X qPCR Master Mix (KAPA Biosystems). All experiments were performed in at least 3 biological replicates. Statistical analysis was performed by Student's *t*-test (compared with vehicle treatment). Primer sequences used for RT-qPCR are available upon request.

### Chromatin immunoprecipitation assays

ChIPs were performed as described before [25]. Briefly, T47D cells were grown for 3 days in RPMI/charcoal-treated fetal calf serum and then treated with 10 nm R5020 for 1 hr. Fixation with 1% formaldehyde proceeded for 10 min at 37°C and was stopped by the addition of glycine to a final concentration of 0.125 M. Cells were harvested, resuspended in lysis buffer (50 mM Tris-HCl pH 8, 10 mm EDTA, 1% sodium dodecyl sulfate) and fragmented chromatin (500–1000 bp) was generated through sonication (Misonix sonicator). Samples were diluted in ChIP dilution buffer (1.2 mM EDTA; 167 mM NaCl; 16.7 mM Tris-HCl, pH 8; 1.1% Triton X-100; and 0.01% SDS), precleared for 2 hr at 4°C with protein A+G magnetic beads (Life Technologies) and used for the ChIP assays with the addition of 5 µg of antibody. The next day the recovered immunocomplexes were washed with the following buffers: washing buffer I (2 mM EDTA; 20 mM Tris-HCl, pH 8.0; 0.1% SDS; 1% Triton X-100; 150 mM NaCl), washing buffer II (2 mM EDTA; 20 mM Tris-HCl, pH 8.0; 0.1% SDS; 1% Triton X-100; 500 mM NaCl), washing buffer III (1 mM EDTA; 10 mM Tris-HCl, pH 8.0; 1% Nonidet P-40; 1% Deoxycholate; 0.25 mM LiCl) and TE (1 mM EDTA, 10 mM Tris-HCl, pH 8.0). After elution, cross-linking was reversed by an overnight incubation at 65°C, samples were incubated with proteinase K and DNA was extracted with phenol-chloroform and EtOH precipitation. Samples were analyzed by qPCR and their enrichment over input was calculated by the 2^−ΔCt^ method after correcting for the IgG negative controls. All experiments were performed in 2–4 biological replicates. Statistical analysis was performed by Student's *t*-test. Primer sequences used for ChIP-qPCR are available upon request.

### Library preparation for next-generation sequencing experiments

For RNA-sequencing, total RNA was extracted from T47D cells using the Trizol reagent (Life Technologies) and was treated with Turbo DNase I (Ambion) for 30 min at 37°C. poly(A)^+^ RNA was isolated using oligo(dT)-conjugated magnetic beads (FastTrack MAG mRNA Isolation Kit, Life Technologies). Library preparation was performed with the ScriptSeq kit (Epicentre) according to the manufacturer's instructions. Briefly, poly(A)^+^ RNA was fragmented at 90°C for 6 min and was, subsequently, subjected to cDNA synthesis. cDNA was tagged at the 3' end, purified using the Agencourt AMPure XP system (BeckmanCoulter), and it was then converted to double-strand cDNA. This product was PCR-amplified for 11 cycles; during this step completion of the addition of the Illumina adaptor sequences and incorporation of an index (ScriptSeq Index PCR Primers, Epicentre) was performed. The PCR product was treated with Exo I for 15 min at 37°C and was purified as described above.

For ChIP-sequencing, DNA was immunoprecipitated from ∼20 million cells, grown and treated as described above, and then it was purified and sonicated to ∼400 bp fragments using the Bioruptor (Diagenode). Fragmented DNA was used for library construction using the NEBNext ChIP-seq sample Prep Master Mix Set 1 (New England Biolabs) and following the manufacturer's instructions. Briefly, this product underwent end repair, dA-tailing and adaptor ligation using the Illumina specific adaptors. In-between enzymatic steps the samples were purified using Agencourt AMPure XP system (BeckmanCoulter). The libraries were PCR-amplified for 11–14 cycles using Phusion HotStart DNA polymerase.

One µl of each library was run on the Bioanalyzer to assess library quantity and quality. Libraries were run on Illumina HiSeq 2000 using 50-bp paired-end sequencing and following standard protocols.

### Next-generation sequencing data analysis and differentially expressed gene (DEG) testing

50-bp paired-end sequencing was performed for PR-immunoprecipitated DNA from R5020-treated cells, for genomic input DNA and for mRNA isolated from progestin- and vehicle- treated cells. The FASTQC package (http://www.bioinformatics.babraham.ac.uk /projects/fastqc/) was used to assess quality of reads. When necessary, Trimmomatic v.0.3 [26] was used to trim reads by applying the parameters LEADING:3 TRAILING:3 SLIDINGWINDOW:4:15 MINLEN:36.

For ChIP-seq data analysis, reads were mapped to the human genome (hg19) using Bowtie v.1.1.2 (default parameters, hg19) [27]. SAM tools [28] were used to select for high quality, uniquely mapped, paired-end reads leading to 14,148,482 total reads. MACS [29] was used for peak calling with default parameters (fold enrichment ≥10 and p-value≤10^-5^) using an equal number of reads from input DNA as a control. For RNA-seq data analysis, after quality filtering, reads were aligned to the UCSC hg19 reference genome using TopHat2 allowing up to two mismatches and discarding reads mapping at multiple locations [30]. This led to the generation of 76,118,132 and 73,174,398 unique paired-end reads for EtOH- and R5020- treated cells respectively. Transcript abundance was quantified using Cufflinks 1.2.1. [31] and the iGenomes Ensembl GTF annotation file as a reference. The normalized expression level of each transcript was measured by FPKM (Fragments Per Kilobase of transcript per Million mapped reads). A threshold of 10 mapped reads was used to define detection at the gene level.

For subsequent analyses we considered the information both at the gene and transcript level. Differentially expressed genes and transcripts were called using Cuffdiff 2 that utilizes Student's t-test to determine if two datasets are different from each other [32]. By default, Cuffdiff 2 generates the lowest p-value to be 5×10^−5^. Genes and transcripts showing an FPKM equal or higher than one at least under one condition (progestin- or vehicle- treated) were retained for further analysis. A threshold of 1.5 was applied on the fold change. Given that the RNA-Seq DEG algorithms generally result in much higher adjusted p-values (0.03∼0.12) than their microarray counterparts (<0.01) [33], and based on the fact that several previously identified PR-regulated genes were listed in our data with p-values up to 0.38, we decided to test higher p-values as cut-offs for the identification of PR-regulated genes. Extensive validation of our data by RT-qPCR led us to finally use a p-value cut-off of 0.15. Using higher p-values led to an increase in the number of false positives. For differentially expressed transcripts the p-value cut-off was ≤0.05. Further manipulation of the data was done with in-house scripts. All datasets have been deposited to GEO (accession number GSE51428).

### Gene Ontology analysis

For functional enrichment analysis of the differentially expressed genes (DEGs) the module FatiGO [34] of the Babelomics bioinformatics suite [35] and the DAVID functional annotation tools [36] were used. Both algorithms use Fischer's exact test to check for significant over-representation of Gene Ontology (GO) annotations, but differ to the gene reference background they use. FatiGO compares DEGs with respect to the whole human genome, while DAVID is more conservative and uses genes associated with terms in the corresponding annotation categories as the reference background.

## Results and Discussion

### Characterization of the RNA-sequencing data

In order to determine the PR-regulated transcriptome in the breast cancer milieu, mRNA was isolated from T47D cells and was subsequently subjected to 50-bp paired-end sequencing. Reads were aligned against the UCSC hg19 reference genome using TopHat 2 [30] and transcript assembly of the aligned reads was performed using Cufflinks [31] and the Refseq database for reference gene annotation (see Materials and Methods).

**Figure 1:**
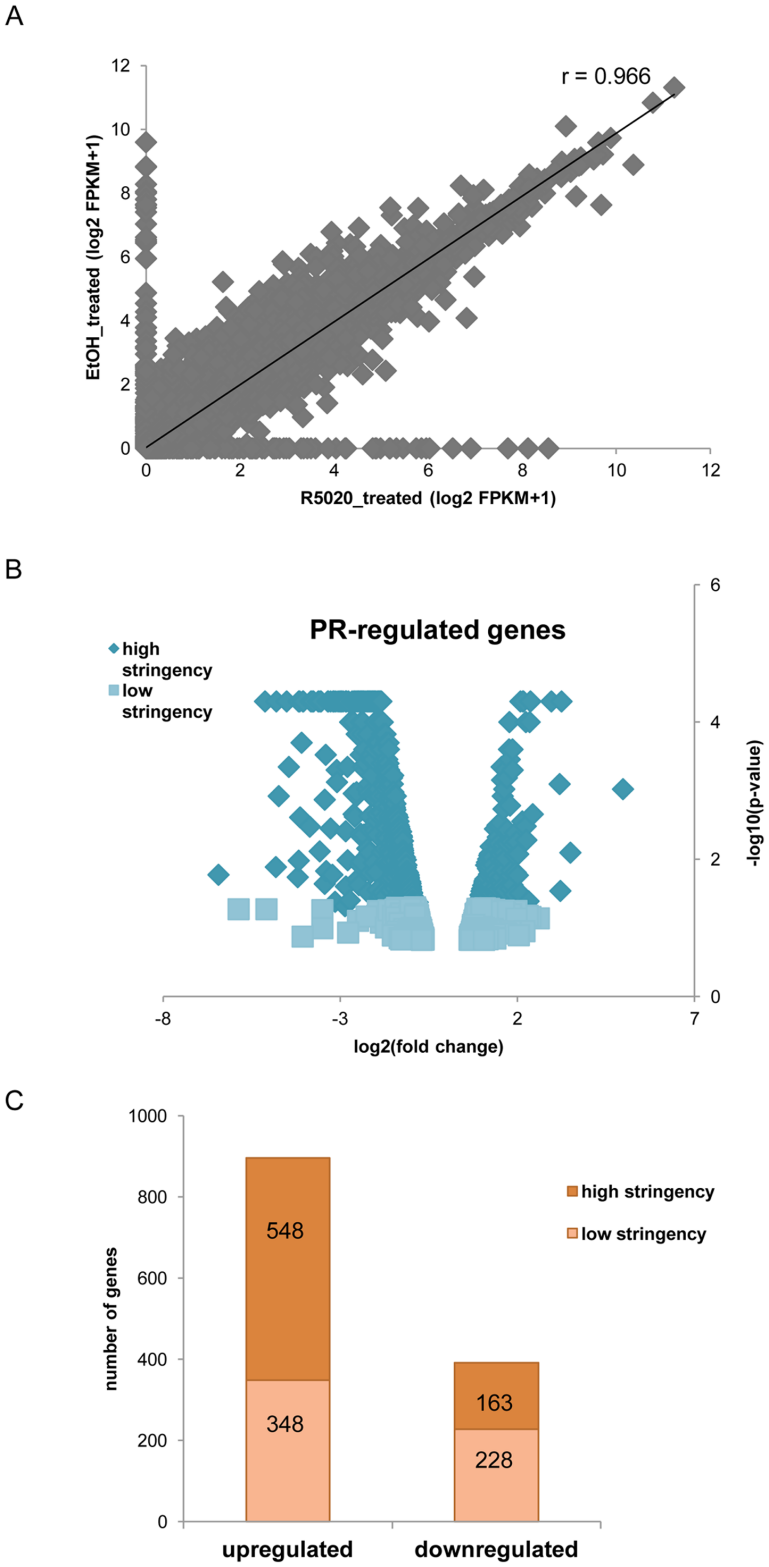
Identification of PR-regulated genes by RNA-sequencing. (A) Scatter plot of global gene expression in the EtOH- and R5020- treated T47D cells. In total, 10,997 and 10,930 genes (FPKM>1) are expressed respectively. The high Pearson correlation coefficient (r = 0.966) indicates similar expression profiles between the two samples as expected. (B) Volcano plot (p-value vs. fold change of expression) for all differentially expressed genes (DEGs) between vehicle- and progestin- treated cells. To determine DEGs, a threshold of 1.5-fold change was set and p-value cut-offs were 0.05 and 0.15 for the high (dark blue) and low (light blue) stringency groups respectively. (C) A total of 711 up-regulated and 576 down-regulated genes were identified and classified to two stringency groups, as described above.

To evaluate our data, we performed an initial analysis on the gene level. The global profiles of gene expression between the two samples were highly correlated with the Pearson correlation coefficient being 0.97 (**Figure 1A**). Among the top 100 most highly expressed genes (data not shown), we identified the expected housekeeping ones (e.g. *GAPDH*, *PPIA* and *TUBA1B*) and several genes associated with the healthy (e.g. *PRLR*), or the neoplastic mammary tissue (e.g. *PIP*, *KRT19* and *MUC1*) [37], [38], [39]. Also included in this list there were members of the heat shock protein 90 family (*HSP90-AA1*, *-AB1* and *-B1*), known molecular chaperones of SHRs [40], and several proto-oncogenes, such as *AGR2* [41], *RAC1* [42] and *GNAS* [43]. Notably, we also found very highly expressed the guanine nucleotide binding protein (G protein), beta polypeptide 2-like 1 (*GNB2L*), calnexin (*CANX*), calreticulin (*CALR*) and beta-2 microglobulin (*B2M*); these are all genes associated with the breast cancer phenotype and they were among the most highly expressed in all breast tumors assayed by RNA-sequencing in a recent study [44].

In overall, the above findings validate our RNA-sequencing data including the transcript assembly and the calculation of transcript abundance as FPKM values.

### Identification and validation of differentially expressed genes

Gene expression microarray experiments are commonly performed after 6–24 hr of progestin treatment and inevitably identify not only primary but also secondary PR targets. The greatly enhanced sensitivity and accuracy delivered by deep-sequencing allowed us to use a shorter treatment period (3 hr) to enrich the differentially regulated genes we identify for primary PR targets. The transcriptomes of the progestin- and vehicle- treated cells were compared using Cuffdiff 2 [32], a differentially expressed genes algorithm. In total, we identified 1287 DEGs and a detailed list of them is provided in **Table S1**. The log2(fold change) of the ratio of EtOH-treated to R5020-treated is given. We set two stringency thresholds to classify all DEGs (see Materials and Methods). The high stringency group included 711 genes and the low stringency group included 576 genes (**Figure 1B**). The majority of the PR targets were up-regulated after progestin treatment (896 genes), while 391 were down-regulated (**Figure 1C**).

**Figure 2:**
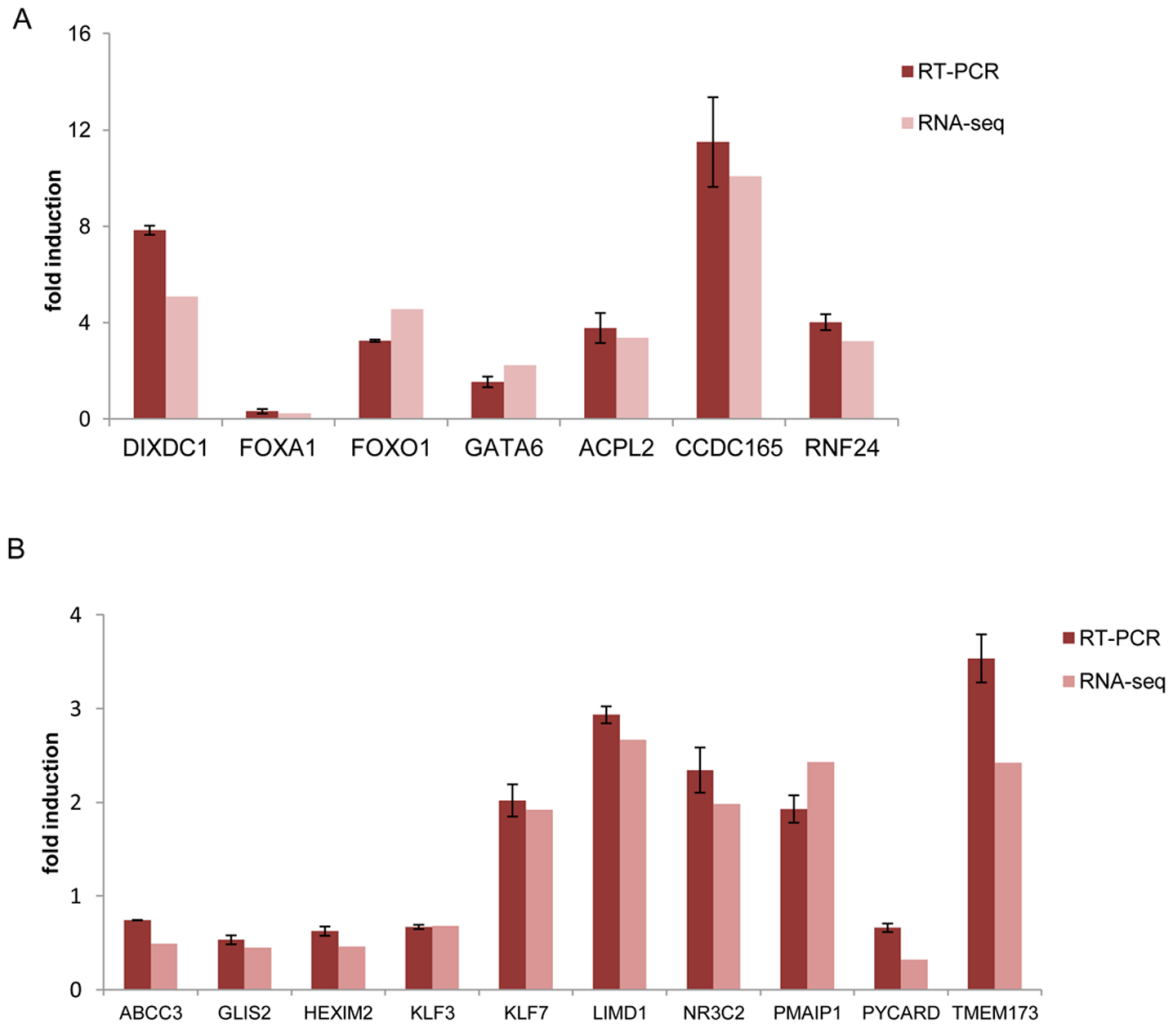
Differentially expressed genes detected by RNA-seq are validated by RT-qPCR. T47D cells were treated for 3-qPCR using specific primers for the genes shown. Expression levels were normalized to GAPDH. A side-by-side comparison of the RT-qPCR results with the RNA-seq data is shown. (A) Expression levels of high stringency genes as measured by RT-qPCR and RNA-seq. *FOXA1* was the only selected down-regulated gene and its expression pattern was confirmed. (B) Expression levels of low stringency genes as measured by RT-qPCR and RNA-seq. Five downregulated (*ABCC3, GLIS2, HEXIM2, KLF3* and *PYCARD*) and five upregulated genes were selected and confirmed by RT-qPCR. Error bars represent the SEM.

To validate our RNA-seq experiments and data analysis, the expression levels of several DEGs from both stringency groups were assayed by RT-qPCR and a side-by-side comparison of these results with the RNA-seq data is shown in **Figure 2**. All genes examined were found to be progestin-regulated in agreement with the RNA-seq data. In **Figure 2A**, we present the results from the high stringency genes. We examined a larger number of genes from the low stringency group (**Figure 2B**) to ensure that we did not include false positives in our analysis. We observed from our RNA-seq data analysis that most genes in the low stringency group were downregulated (**Figure 1C**), thus we selected to examine an equal number of induced and repressed genes from this group (**Figure 2B**). For two of these genes, *ABCC3* and *PYCARD*, the degree of downregulation was not accurately measured by RNA-seq, but in overall, changes in expression levels detected by the two techniques were very similar for most genes tested providing additional support for the accuracy of the RNA-seq data.

We also compared our dataset with several other previously published datasets of PR-regulated genes generated by microarray expression experiments in T47D cells [10], [11], [12], [13], [14], [15]. We found that both stringency groups contained previously reported PR-regulated genes (350 out of the 1287) (**Table S1**).

The above data, taken together, further confirm the validity of our RNA-seq data and analysis and lead to the identification of hundreds of new PR targets.

### Early progestin-induced gene expression changes in breast cancer cells

**Figure 3:**
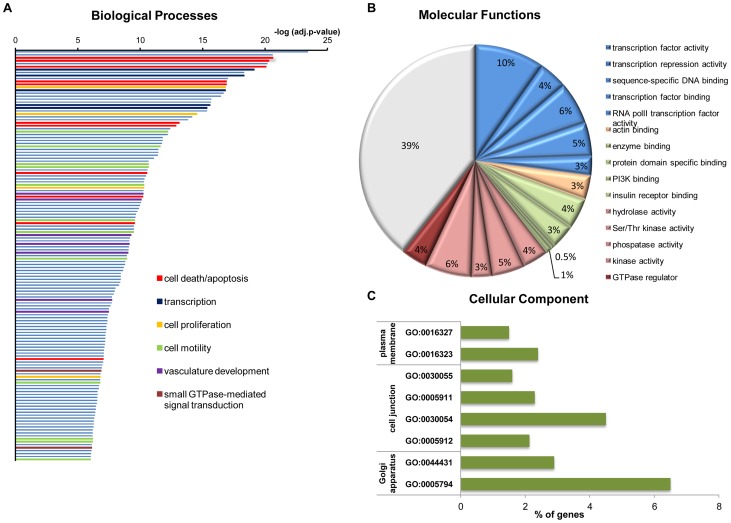
Gene ontology analysis for PR target genes. (A) Highly enriched biological processes were determined by using FatiGO. All GO annotations related to the same biological process are shown in the same color. Grey bars represent other processes. (B) Highly enriched molecular functions were determined by using FatiGO and the top fifteen are presented here. Results are clustered by function and the percentage of DEGs that were associated with each GO annotation is shown. (C) Eight of the top ten most highly enriched GO annotations for cellular component (as determined by FatiGO) fall into 3 broad categories: plasma membrane, cell junction and Golgi apparatus. Percentage of DEGs that were identified in each category is shown.

To gain some insight into the biological significance of our data, we performed gene ontology analysis for all DEGs using the tools FatiGO [34] and DAVID [36]; both algorithms yielded similar results. GO analysis was organized around the three basic "principles" of biological process, molecular function and cellular component (**Figure 3**).

Key biological processes associated with breast cancer initiation and progression, such as regulation of transcription, apoptosis and cell proliferation, were found to be highly enriched among PR targets (**Figure 3A**). The DEGs involved in regulation of cell death are listed in **File S1, table A**. Interestingly, they include an, approximately, equal number of genes that promote or suppress apoptosis (**Figure S1**) in accordance with the dual role of PR in cell proliferation [45]. Right after progestin treatment T47D cells go through a proliferative phase followed by a second phase of growth inhibition [45]. Our data show that early transcriptional responses are not restricted to cell proliferative genes, but also include induction of genes with apoptotic effects. This suggests that the PR initiates simultaneously a proliferative and an anti-proliferative program and the decision which one will prevail, probably, depends upon the specific cell context. These findings fit with the hypothesis by Lange et al that progesterone acts as a priming agent that induces cellular changes that permit other factors to influence the ultimate proliferative or differentiative state of the cells [46].

The most numerous target group (258 out of 1200 genes analyzed by DAVID) is comprised of genes playing a role in regulation of transcription (**File S1, table B**). It includes members of principal families of transcription factors, such as the GATA, FOX and E2F families that control a wide spectrum of biological functions. Deregulation of the expression of these factors has a crucial role in the development and progression of cancer [47], [48], [49]. Another example is the family of the Krüppel-like factors (KLF) with most of its members being under progestin regulation in T47D cells (**Table S1**). Our data confirm previous findings that several KLFs are PR targets (see **Table S1**) and they add to this list *KLFs 3*, *6*, and *7* (**Table S1 and** **Figure 2B**). Recent studies have documented KLF family members in the control of cell proliferation, differentiation, and apoptosis in steroid-responsive mammary and uterine endometrial cells [50]; some of them have been implicated in breast cancer progression [50]. It is reasonable to assume that cross-talk between KLF and PR signaling may partly mediate the receptor's effects on these processes. Deregulation of PR signaling may lead to aberrant expression of KLFs and loss of control over these critical events in breast cancer.

In accordance with the above analysis, we, also, find that the most highly enriched molecular functions in the DEGs are related to transcription factor activity (blue sectors in **Figure 3B**), supporting the notion that the PR initiates a new transcriptional program in the cell as a response to the external stimulus.

Other biological pathways that are overrepresented are related to cell motility and angiogenesis (**Figure 3A**), which are key steps in cancer cell growth and metastasis. Shortly after progestin treatment the expression status of several genes involved in cell motion (**File S1, table C**) is affected substantiating previous reports of progestin-induced breast cancer cell migration and invasion [51], [52]. Moreover, a significant number of PR targets are components of cell junctions (cell to cell and cell to extracellular matrix) (**Figure 3C**) confirming that the receptor plays an important role in regulating cell motion. Our data also offer new insight into the role of PR in angiogenesis by identifying dozens of target genes involved in this process (**File S1, table D**). *VEGFA*, a potent angiogenic factor that stimulates breast cancer cell growth *in vitro* and *in vivo* [53] and *THBS1*, an inhibitor of angiogenesis that promotes tumor progression and metastasis [54], had been reported before as PR-regulated genes [55], [56], [57], [58]. We find that other well-known angiogenic factors, such as *ACVR1* and *EDN1* are, also, under progestin control (**File S1, table D**). These data strongly suggest that PR-orchestrated networks are involved in cell motility and vasculature development in breast cancer cells. It is plausible that aberrant PR signaling in breast tumors may be a contributing factor to tumor vascularization and metastasis.

Notably, the same functional categories are also enriched when examining the high stringency group alone, confirming the validity of our differential gene analysis (data not shown).

### Components of the small-GTPase signaling pathways are direct PR targets

The GO analysis described above revealed that a significant fraction of PR targets is involved in "small-GTPases mediated signal transduction" (**Figure 3A**), most likely functioning as "GTPase regulators" (**Figure 3B**). This was a novel finding that raised our interest, since deregulation of small-GTPases signaling may play a role in cancer progression [23]. The small GTPases are small G-proteins that can bind and hydrolyze GTP, cycling in this way between an inactive (GDP-bound) and an active (GTP-bound) state. Their activity is regulated by GTPase activating proteins (GAPs) and guanine nucleotide exchange factors (GEFs). They are localized to multiple membrane compartments including the plasma membrane and the Golgi apparatus and this is, probably, the reason behind the significant number of PR targets associated with these cellular components (**Figure 3C**).

**Figure 4:**
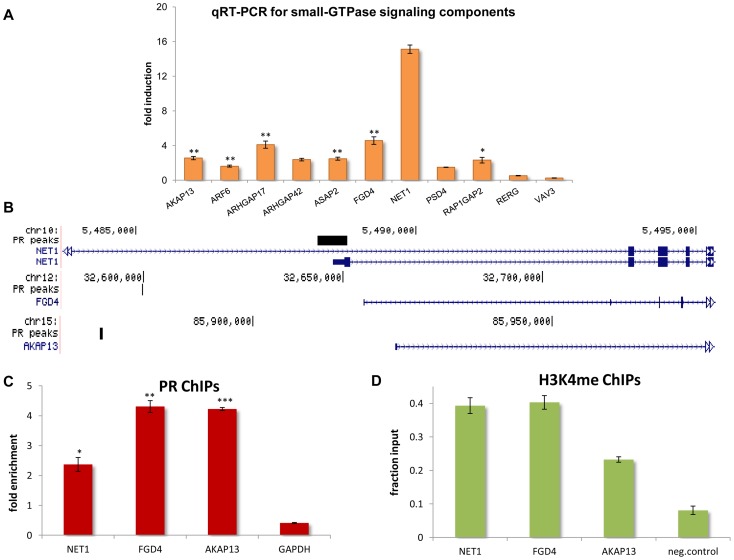
Components of small-GTPases signaling pathways are direct PR targets. (A) T47D cells, treated with R5020 or EtOH for 3 hrs, were used for RNA extraction. RT-qPCR was performed using specific primers for two small GTPases (*ARF6, RERG*) and several GEFs (*AKAP13*, *FGD4, NET1, PSD4, VAV3*) and GAPs (*ASAP2*, *ARHGAP17, ARHGAP42, RAP1GAP2*). Expression levels were normalized to GAPDH. Error bars indicate the SEM. (p-value<0.005, single asterisk indicates p-value<0.5 and double asterisk p-value<0.05) (B) Visualization of the ChIP-seq data on the UCSC browser. PR binding sites in an intron of the *NET1* gene and in distal intragenic regions of *FGD4* and *AKAP13* are depicted by the black blocks. (C) ChIP-qPCR experiments show increased PR binding in the three sites shown in (B) after 1 hr of progestin treatment (single asterisk indicates p-value<0.05, double asterisk p-value<0.01 and triple asterisk p-value<0.001). The promoter of GAPDH is used as a negative control. (D) ChIP-qPCR experiments for methylated H3K4. The PR binding sites shown in (B) are also enriched for this mark (p-value<0.05) of active enhancers and promoters. An intergenic region is used as a negative control. Error bars represent SE.

Functional annotation using FaTIGO [34] and DAVID [36] and manual inspection of all DEGs identified 74 genes in total involved in small-GTPases signal transduction. The vast majority of them (59 of 74) are small-GTPases that belong to the Ras superfamily or they are regulators of these enzymes (**Table 1**). Several of these genes were experimentally validated by RT-qPCR (**Figure 4A**). Three hours after progestin treatment all genes assayed were significantly up-regulated, except *RERG* and *VAV3* that were repressed in agreement with the RNA-seq data (**Table S1**).

**Table 1:** Progestin-regulated small-GTPases, GAPs and GEFs.

**Ras family**		
**Small GTPases**	**GAPs**	**GEFs**
LRRK2	RASA2	PLCE
RASL10B	RASA3	RASGEF1A
RERG	RASA4	RASGRP1
RRAS2	RASAL1	SOS1
	SIPA1	
**Rho family**		
**Small GTPases**	**GAPs**	**GEFs**
RHOB	ARHGAP17	AKAP13
RHOBTB2	ARHGAP23	ARHGEF2
RHOU	ARHGAP26	ARHGEF26
RHOV	ARHGAP32	ARHGEF37
RND1	ARHGAG40	FGD3
	ARHGAP42	FGD4
	SRGAP1	ITSN1
	SRGAP2	NET1
	SRGAP3	PLEKHG6
	SRGAP2B	SPATA13
	STARD13	VAV3
**Arf family**		
**Small GTPases**	**GAPs**	**GEFs**
ARF6	ASAP2	CYTH1
ARL4C		IQSEC1
ARL4D		PSD4
ARL5B		
**Rab family**		
**Small GTPases**	**GAPs**	
RAB12	TBC1D12	
RAB33B	TBC1D20	
RAB4A	TBC1D4	
RAB9B		
**Rap family**		
**Small GTPases**	**GAPs**	**GEFs**
RAP2B	RAP1GAP2	RAPGEF6
RAP2C		

The above data demonstrate that several small-GTPases, GAPs and GEFs are early (immediate) PR targets. Next, we asked whether these genes were, also, direct PR targets, where the receptor directly controlled their expression by binding to regulatory sequences. To this end we performed ChIP experiments with an antibody against the PR followed by paired-end NGS to accurately map the receptor's binding sites. In overall, we identified 11,180 PR binding sites in treated T47D cells. Validation of these data is shown in **Figure S2**. *FKBP5* is a well-known PR target and various PR binding sites have been reported in a distant intron of the human locus and in the first intron of the mouse one [25], [59]. Our ChIP-seq experiments identified a number of new PR binding sites several kilobases upstream of the *FKBP5* transcript variant 1 (NM_004117) (**Figure S2A**), which is the PR-regulated transcript (**File S2, table A_DETs**). These sites were all verified by ChIP-qPCR (**Figure S2B**). We also assayed by ChIP-qPCR several other PR binding sites associated with PR-regulated genes and they were all found to be enriched for receptor binding (**Figure S2C**).

Examination of our ChIP-seq dataset indicated that the majority of small GTPases, GAPs and GEFs (34 out of 59) were indeed associated with a PR-binding site within 50 Kb from the gene locus. Since the Rho GEFs are overrepresented among the PR targets listed in **Table 1**, we selected three of them (*NET1*, *FGD4* and *AKAP13*) for further analysis. Our ChIP-seq data revealed a PR binding site in an intron of the *NET1* gene and in distal intergenic regions ∼50 Kb up-stream of the transcription start sites of *FGD4* and *AKAP13* (**Figure 4B**). ChIP-qPCR experiments confirmed that these sites were enriched for PR binding after progestin treatment (**Figure 4C**). To further show that receptor recruitment on these sites is not a random event, but it has a functional role, we performed ChIPs for H3K4me, a mark for promoters and enhancers [60]. We found that these sites were enriched for this histone modification (**Figure 4D**) strongly arguing in favor of their regulatory role.

Taken together the above data suggest that the PR can modulate the expression of a large number of small-GTPases and associated regulators. Several of them appear to be direct PR targets as their mRNA levels are affected shortly after progestin treatment and following PR binding in regulatory sites. Most PR targets are Ras and Rho small-GTPases regulators (GAPs and GEFs) (**Table 1**). The Ras family members are involved in control of cell proliferation, while the Rho GTPases play an important role in cytoskeleton organization [23], and as such they are involved in cell adhesion, migration, proliferation, survival, differentiation and malignant transformation [61]. We confirmed that several Rho GEFs are under direct PR regulation (**Figures 4A and 4C**). It is plausible that, in the breast cancer milieu, the intensified progestin stimulus induces over-expression of Rho GEFs leading to aberrant activation of the cognate small-GTPases, which, in this way, mediate hormonal control over important biological processes. In agreement with this hypothesis, the most common cause of aberrant small-GTPase signaling in cancer is the altered expression or activation of their regulators [61].

Notably, it has been shown that non-genomic actions of the ligand-activated PR lead to activation of the RhoA/Rho-associated kinase (ROCK-2) cascade in T47D cells [52], [62]. This signaling pathway, ultimately, directs remodeling of the actin cytoskeleton and formation of membrane ruffles required for cell movement [52]; it, also, leads to rapid activation of the focal adhesion (FA) kinase and increased formation of FA complexes, which provide anchoring sites for cell attachment to the extracellular matrix during cell movement and invasion [62]. During preparation of this manuscript a study came out that described the PR-targetome during mammary gland branching morphogenesis [63]. Interestingly enough, the authors, also, identified components of "small-GTPases mediated signal transduction" to be highly enriched among the target genes [63]. They went on to show that progesterone activation of Rac (a Rho small-GTPase) signaling is necessary to induce side-brunching [63]. These findings are in agreement with our own data highlighting the small-GTPases signaling pathways as progestin-regulated networks in healthy and malignant mammary cells.

### PR dictates alternative promoter use decisions in breast cancer cells

For the identification of differentially expressed transcripts (DETs) after progestin stimulation, we used Cuffdiff 2 [32] as described before. We set more stringent parameters for data analysis on the transcript level (≥1.5-fold change of expression and p-value≤0.05) leading to the identification of 1014 DETs (data not shown). To ensure the validity of our findings, we limited further analysis to the top 80 DETs (p-value≤5×10^−5^) (**File S2, table A_DETs**). Thirty of them were generated by genes that had multiple transcripts in the RefSeq database [64] and they are listed in **File S2, table B_annotated transcripts**. Annotation of the transcript variants (columns B and C in Suppl. file 3, sheet 2) was done according to the RefSeq database. The protein isoforms they encode are denoted as "canonical" according to the Uniprot database (column D). Information from both the RefSeq and the Uniprot databases was used in order to mark protein isoforms as "distinct" (different than the canonical) or "not distinct" (same as the canonical) (column D). Transcript variants generated by alternative splicing or alternate promoter usage relative to the canonical isoform are denoted as AS or AP respectively (column D). There is an equal number (12) of PR-regulated transcript variants that are generated either by AS or by AP, while 6 of them use both mechanisms (column F). Interestingly, the PR-regulated transcript variants of 26 out of the 29 genes (1 gene encodes for non-coding RNAs) encode for distinct protein isoforms (columns D and F), suggesting that progestin signaling may contribute significantly to the proteomic diversity of the cell.

**Figure 5:**
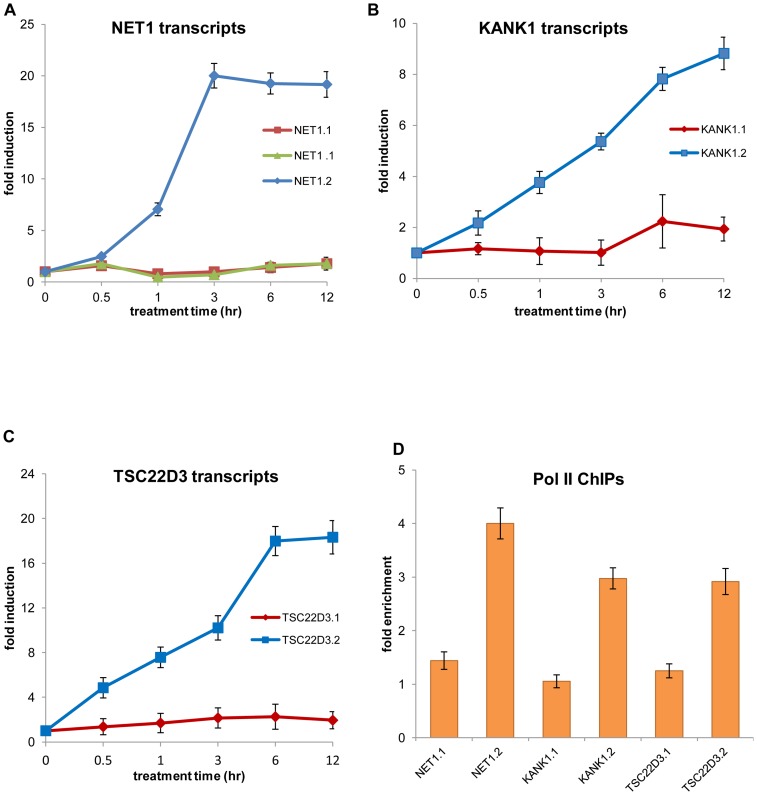
PR regulation of specific transcript variants. T47D cells were treated for 0 to 12-qPCR using transcript-specific primers. Expression levels were normalized to GAPDH. (A) Time course experiments for the *NET1* transcripts show strong induction of the NET1.2 variant after progestin treatment, while the NET1.1 variant retains a stable expression pattern. To confirm the NET1.1 levels of expression, two different set of transcript-specific primers were used (in green and red) giving identical results. (B) Time course experiments for the two *KANK1* transcripts confirm upregulation of the KANK1.2 after progestin treatment. (C) Time course experiments for the two TSC22D3 transcript variants show that only the TSC22D3.2 variant is strongly upregulated after progestin treatment. (D) ChIP-qPCR experiments show significant increase in polII binding on the promoters of NET1.2, KANK1.2 and TSC22D3.2 after 1 hr of progestin treatment (ethanol-treated control is set to 1) (p-value<0.05). Error bars represent SE except in (A), where they represent the SEM.

Eight (*ARID5, GREB1, KANK1, NET1, PFKB3, RCAN1, TIPARP* and *TSC22D3*) of these 30 genes had two or more transcript variants expressed in vehicle-treated T47D cells (data not shown), however, only one of these variants was differentially regulated after progestin treatment (**File S2, table A_DETs**). To confirm these results, we examined the 3 genes (*NET1, KANK1* and *TSC22D3*) whose transcript variants appeared to be expressed in, approximately, similar levels in vehicle-treated T47D cells (**Table 2**). We designed variant-specific primers and we performed a time-course RT-qPCR study; the results are shown in **Figure 5**. For all three genes, transcript variants 1 (herein called NET1.1, KANK1.1 and TSC22D3.1) retained a, relatively, stable level of expression, while transcript variants 2 (herein called NET1.2, KANK1.2 and TSC22D3.2) were strongly induced shortly after progestin treatment (**Figures 5A–C**). This is in absolute agreement with the RNA-seq data (**Table 2**). Given that an older study had found that both NET1 transcripts were up-regulated after progestin treatment [22], we used two different sets of primers for the NET1 transcript variant 1 (NET1.1) and we repeated the experiment in 3 biological replicates; both primer sets gave identical results in all three experiments (**Figure 5A**).

**Table 2:** Expression levels (in FPKM) of transcript variants according to the RNA-seq data.

Official gene symbol	GenBank accession ID	R5020 (FPKM)	EtOH (FPKM)	log2(fold_change)
**NET1**				
NET1 transcript variant 1	NM_001047160	10.0867	8.66451	−0.219266
NET1 transcript variant 2	NM_005863	118.522	5.59903	−4.40383
**KANK1**				
KANK1 transcript variant 1	NM_153186	12.1742	1.73468	−2.81108
KANK1 transcript variant 2	NM_015158	1.87683	2.07454	0.14449
**TSC22D3**				
TSC22D3 transcript variant 1	NM_198057	2.64031	2.02448	−0.383154
TSC22D3 transcript variant 2	NM_004089	38.2182	4.95535	−2.9472

We further investigated the mechanisms that mediated this preferential transcript regulation. In all three genes, the alternative transcripts were generated by alternative promoters. In the case of *KANK1* and *NET1*, our ChIP-seq experiments had revealed PR-binding sites in the first intron of KANK1.2 (not shown) and in the promoter of NET1.2 (**Figure 4B**); these sites were confirmed by ChIP-qPCR experiments (**Figures S2C and 4C** respectively). For the *TSC22D3* gene we did not find any PR-binding sites within a 50Kb distance. It is possible that our ChIP-seq experiments failed to capture such a site. It is also possible that the PR regulatory site is located in a greater distance or that the induction of TSC22D3.2 is not a direct transcriptional effect of the receptor. Since *TSC22D3* is a known glucocorticoid-regulated gene with well-characterized GREs [65], we designed primers encompassing the GR-binding site and performed ChIP-qPCR for the PR. We did not find any enrichment for PR-binding in this region (data not shown). Subsequently, we performed polII ChIP-qPCR experiments and we showed that polII recruitment on the NET1.2, KANK1.2 and TSC22D3.2 promoters was increased in response to progestin, but it was not, significantly, affected on the promoters of the non-progestin regulated variants (**Figure 5D**). Assumingly, PR recruits transcription co-factors that facilitate polII loading onto the regulated promoters. Communication of the PR complex with the non-regulated promoters may be prevented through binding of the insulator factor CTCF, as it has been suggested for the differential regulation of transcript variants by the estrogen receptor [22].

These findings are of particular importance, since, oftentimes, transcript variants of the same gene encode for different protein isoforms with diverse functions. A striking example is provided by the *NET1* gene itself. NET1 is a RhoA specific GEF. RhoA is aberrantly expressed in many human cancers, including breast cancer, and its activation is essential for cancer cell migration and invasion [66]. The two transcript variants generated by the *NET1* gene (**Figure 4** **B**), a long one known as NET1 (NET1.1, NM_001047160) and a shorter one also known as NET1A (NET1.2, NM_005863), encode for isoforms with potentially different functions [22]. Studies have indicated that the NET1 isoform is important for cell proliferation [22], while the NET1A controls cytoskeleton reorganization and cell motility [22], [66], [67]. Specifically, NET1A is required for FAK activation, focal adhesion maturation and it is necessary for amoeboid extracellular matrix invasion of breast cancer cells [66]. It is, therefore, possible that NET1A plays an important role in metastatic breast cancer. According to our data PR induces over-expression specifically of the NET1A (NET1.2) variant. It is tempting to speculate that increased levels of NET1A may lead to aberrant activation of RhoA and mediate breast cancer metastasis.

The transcript variants generated by *KANK1* and *TSC22D3*, also, encode for distinct protein isoforms (**File S2, table B_annotated transcripts**) with, potentially, different functions. *KANK1* was first identified as a candidate tumor suppressor gene for renal cell carcinoma and it has several alternative promoters, by which different types of transcripts are generated from the same locus (reviewed in [68]). It encodes an ankyrin-repeat domain-containing protein, which, negatively, regulates the formation of actin stress fibers and cell migration through inhibition of RhoA, while it may also have a growth inhibitory effect [68]. Two types of KANK1 proteins have been reported with distinct tissue distribution [69], but they have not been functionally characterized. *TSC22D3* (also known as *GILZ*) is a well-studied glucocorticoid-regulated gene in the immune system. Four different isoforms have been identified and characterized; these are not functionally redundant, but rather, they are involved in distinct aspects of cellular physiology and modulate distinct signaling pathways [70]. The PR-regulated variant (NM_004089) encodes for isoform GILZ1 [70]. GILZ1 appears to be the most potent isoform in stimulation of Na^+^ transport and repression of NF-κB. Few studies have addressed the role of GILZ in cancer. A recent study showed that it was expressed in epithelial ovarian cancer, where it increased tumor cell proliferation, activated AKT and regulated p21 and cyclin D1 expression, events that are associated with tumor progression [71].

Taken together, the above data strongly suggest that the PR can dictate promoter use decisions leading to significant expression changes of specific transcript variants. Future studies should aim to characterize these variants, examine their potential clinical value in hormone responsive breast tumors and, more importantly, determine the functional properties of the isoforms they encode.

## Conclusions

The era of next-generation sequencing has immensely advanced our understanding in nuclear hormone receptor signaling [72]. Several studies were particularly aimed to the estrogen receptor and they offered significant new insights into the complex regulatory gene networks controlled by the receptor in breast cancer cells [73]. However, this powerful technique has not been as extensively used in the study of the PR. A few recent papers have performed genome-wide mapping of PR binding sites in breast cancer cells [74], [75], [76] confirming that the receptor binds more frequently in intra- and inter- genic regions than in the promoters of target genes in agreement with earlier observations [25], [59]. To complement these studies and shed more light into the progestin-regulated gene networks in breast cancer cells, we employed 50bp paired-end sequencing to identify early responsive transcripts. Genes that display expression changes at early time points are more likely to be primary PR targets; however, these changes are usually of small magnitude and are often missed by microarray studies. Our experimental approach provided the necessary depth to detect such changes. We identified 1287 DEGs and we extensively validated new targets by RT-qPCR. Our data offer a new insight into the multifaceted role of PR in breast cancer biology and point to new routes future research can take. For example, we find that the PR alters the expression levels of key transcription factors and, in this manner, it may be affecting important transcriptional networks that govern cell fate. It remains to be seen whether these changes accommodate the needs of the cancer cell and corroborate the role of PR in promoting tumorigenesis. Of particular importance is the finding that the PR regulates a plethora of genes that participate in small-GTPase signaling cascades. Integration of the transcriptome and PR-cistrome profiling of hormonally treated cells strongly suggests that several small-GTPases regulators are direct PR targets. It is likely that progestin modulation of their expression levels leads to deregulation of the respective small-GTPases and the processes they control and eventually contributes to mammary tumorigenesis. Finally, our data reveal that the PR regulates the expression of specific transcript variants, and it, most likely, contributes to a more complex proteomic profile of the breast cancer cell. Future studies will show whether these specific PR-regulated transcripts may have clinical utility in prognosis and/or the development of targeted therapies.

## Supporting Information

Figure S1 **PR-regulated genes involved in cell death/apoptosis.** GO annotation and literature search led to the functional categorization of genes involved in cell death as pro-apoptotic or anti-apoptotic. A few genes were denoted as both pro- and anti- apoptotic and were counted in both groups.
(TIF)

Figure S2 **ChIP-sequencing experiments for the PR identify receptor binding sites in T47D cells.** (A) Cells treated with R5020 for 1 hr were used for ChIP experiments with an antibody against the PR. Immunoprecipitated DNA was used in sequencing experiments and representative data are shown here. Four PR binding sites (depicted by black blocks) were found in distal enhancer elements of *FKBP5* transcript variant 1 (NM_004117), which is the PR-regulated transcript. (B) ChIP-qPCR experiments for the PR were performed in progestin- and vehicle- treated cells. The primers used amplified part of some of the PR binding sites (depicted by red arrows and labeled a-e) identified in the *FKBP5* locus by ChIP-seq. Error bars indicate the SEM. (single asterisk indicates p-value<0.05, double asterisk p-value<0.005 and triple asterisk p-value<0.005). (C) As in (B), but primers used amplified part of the PR binding sites associated with the genes shown. A known PR binding site in the promoter of CDKN1A is used as a positive control and the promoter of GAPDH as a negative one (p-value<0.001, single asterisk indicates p-value<0.05, double asterisk p-value<0.01 and triple asterisk p-value<0.005).
(TIF)

Table S1 **Differentially expressed genes in T47D cells after 3 hours of progestin treatment.** The log2(fold change) of the ratio of EtOH-treated to R5020-treated is given. Genes identified by previous gene expression microarrays studies are accompanied by reference numbers.
(XLSX)

File S1 **Gene Ontology analysis of progestin-regulated genes.**
(XLSX)

File S2 **Differentially expressed transcripts (DETs) in T47D cells after 3 hours of progestin treatment.** The top 80 DETs (p-value≤5×10^-5^) are listed in table A_DETs. Thirty of them are generated by genes that have multiple transcripts in the RefSeq database [64] and they are listed in table B_annotated transcripts.
(XLSX)

## References

[1] Mulac-Jericevic B, Conneely OM (2004) Reproductive tissue selective actions of progesterone receptors. Reproduction 128: 139–146. 15280552 10.1530/rep.1.00189

[2] Conneely OM, Jericevic BM, Lydon JP (2003) Progesterone receptors in mammary gland development and tumorigenesis. J Mammary Gland Biol Neoplasia 8: 205–214. 14635795 10.1023/a:1025952924864

[3] Kim JJ, Kurita T, Bulun SE (2013) Progesterone action in endometrial cancer, endometriosis, uterine fibroids, and breast cancer. Endocr Rev 34: 130–162. 23303565 10.1210/er.2012-1043 PMC3565104

[4] Brisken C (2013) Progesterone signalling in breast cancer: a neglected hormone coming into the limelight. Nat Rev Cancer 13: 385–396. 23702927 10.1038/nrc3518

[5] Chatterton RT Jr, Lydon JP, Mehta RG, Mateo ET, Pletz A, et al (2002) Role of the progesterone receptor (PR) in susceptibility of mouse mammary gland to 7,12-dimethylbenz[a]anthracene-induced hormone-independent preneoplastic lesions in vitro. Cancer Lett 188: 47–52. 12406547 10.1016/s0304-3835(02)00461-5

[6] Lydon JP, Ge G, Kittrell FS, Medina D, O'Malley BW (1999) Murine mammary gland carcinogenesis is critically dependent on progesterone receptor function. Cancer Res 59: 4276–4284. 10485472

[7] Heiss G, Wallace R, Anderson GL, Aragaki A, Beresford SA, et al (2008) Health risks and benefits 3 years after stopping randomized treatment with estrogen and progestin. JAMA 299: 1036–1045. 18319414 10.1001/jama.299.9.1036

[8] Rossouw JE, Anderson GL, Prentice RL, LaCroix AZ, Kooperberg C, et al (2002) Risks and benefits of estrogen plus progestin in healthy postmenopausal women: principal results From the Women's Health Initiative randomized controlled trial. JAMA 288: 321–333. 12117397 10.1001/jama.288.3.321

[9] Obr AE, Edwards DP (2012) The biology of progesterone receptor in the normal mammary gland and in breast cancer. Mol Cell Endocrinol 357: 4–17. 22193050 10.1016/j.mce.2011.10.030 PMC3318965

[10] Ghatge RP, Jacobsen BM, Schittone SA, Horwitz KB (2005) The progestational and androgenic properties of medroxyprogesterone acetate: gene regulatory overlap with dihydrotestosterone in breast cancer cells. Breast Cancer Res 7: R1036–1050. 16457685 10.1186/bcr1340 PMC1410743

[11] Graham JD, Yager ML, Hill HD, Byth K, O'Neill GM, et al (2005) Altered progesterone receptor isoform expression remodels progestin responsiveness of breast cancer cells. Mol Endocrinol 19: 2713–2735. 15976005 10.1210/me.2005-0126

[12] Jacobsen BM, Schittone SA, Richer JK, Horwitz KB (2005) Progesterone-independent effects of human progesterone receptors (PRs) in estrogen receptor-positive breast cancer: PR isoform-specific gene regulation and tumor biology. Mol Endocrinol 19: 574–587. 15563544 10.1210/me.2004-0287

[13] Knutson TP, Daniel AR, Fan D, Silverstein KA, Covington KR, et al (2012) Phosphorylated and sumoylation-deficient progesterone receptors drive proliferative gene signatures during breast cancer progression. Breast Cancer Res 14: R95. 22697792 10.1186/bcr3211 PMC3446358

[14] Richer JK, Jacobsen BM, Manning NG, Abel MG, Wolf DM, et al (2002) Differential gene regulation by the two progesterone receptor isoforms in human breast cancer cells. J Biol Chem 277: 5209–5218. 11717311 10.1074/jbc.M110090200

[15] Wan Y, Nordeen SK (2002) Overlapping but distinct gene regulation profiles by glucocorticoids and progestins in human breast cancer cells. Mol Endocrinol 16: 1204–1214. 12040008 10.1210/mend.16.6.0848

[16] Kornblihtt AR, Schor IE, Allo M, Dujardin G, Petrillo E, et al (2013) Alternative splicing: a pivotal step between eukaryotic transcription and translation. Nat Rev Mol Cell Biol 14: 153–165. 23385723 10.1038/nrm3525

[17] David CJ, Manley JL (2010) Alternative pre-mRNA splicing regulation in cancer: pathways and programs unhinged. Genes Dev 24: 2343–2364. 21041405 10.1101/gad.1973010 PMC2964746

[18] Dutertre M, Vagner S, Auboeuf D (2010) Alternative splicing and breast cancer. RNA Biol 7: 403–411. 20622514 10.4161/rna.7.4.12152

[19] Omenn GS, Menon R, Zhang Y (2013) Innovations in proteomic profiling of cancers: Alternative splice variants as a new class of cancer biomarker candidates and bridging of proteomics with structural biology. J Proteomics 90: 28–37. 23603631 10.1016/j.jprot.2013.04.007 PMC3841011

[20] Pajares MJ, Ezponda T, Catena R, Calvo A, Pio R, et al (2007) Alternative splicing: an emerging topic in molecular and clinical oncology. Lancet Oncol 8: 349–357. 17395108 10.1016/S1470-2045(07)70104-3

[21] Bhat-Nakshatri P, Song EK, Collins NR, Uversky VN, Dunker AK, et al (2013) Interplay between estrogen receptor and AKT in estradiol-induced alternative splicing. BMC Med Genomics 6: 21. 23758675 10.1186/1755-8794-6-21 PMC3687557

[22] Dutertre M, Gratadou L, Dardenne E, Germann S, Samaan S, et al (2010) Estrogen regulation and physiopathologic significance of alternative promoters in breast cancer. Cancer Res 70: 3760–3770. 20406972 10.1158/0008-5472.CAN-09-3988

[23] Csepanyi-Komi R, Levay M, Ligeti E (2012) Small G proteins and their regulators in cellular signalling. Mol Cell Endocrinol 353: 10–20. 22108439 10.1016/j.mce.2011.11.005

[24] Whitman S, Wang X, Shalaby R, Shtivelman E (2000) Alternatively spliced products CC3 and TC3 have opposing effects on apoptosis. Mol Cell Biol 20: 583–593. 10611237 10.1128/mcb.20.2.583-593.2000 PMC85138

[25] Magklara A, Smith CL (2009) A composite intronic element directs dynamic binding of the progesterone receptor and GATA-2. Mol Endocrinol 23: 61–73. 19036901 10.1210/me.2008-0028 PMC2646598

[26] Lohse M, Bolger AM, Nagel A, Fernie AR, Lunn JE, et al (2012) RobiNA: a user-friendly, integrated software solution for RNA-Seq-based transcriptomics. Nucleic Acids Res 40: W622–627. 22684630 10.1093/nar/gks540 PMC3394330

[27] Langmead B, Trapnell C, Pop M, Salzberg SL (2009) Ultrafast and memory-efficient alignment of short DNA sequences to the human genome. Genome Biol 10: R25. 19261174 10.1186/gb-2009-10-3-r25 PMC2690996

[28] Li H, Handsaker B, Wysoker A, Fennell T, Ruan J, et al (2009) The Sequence Alignment/Map format and SAMtools. Bioinformatics 25: 2078–2079. 19505943 10.1093/bioinformatics/btp352 PMC2723002

[29] Zhang Y, Liu T, Meyer CA, Eeckhoute J, Johnson DS, et al (2008) Model-based analysis of ChIP-Seq (MACS). Genome Biol 9: R137. 18798982 10.1186/gb-2008-9-9-r137 PMC2592715

[30] Kim D, Pertea G, Trapnell C, Pimentel H, Kelley R, et al (2013) TopHat2: accurate alignment of transcriptomes in the presence of insertions, deletions and gene fusions. Genome Biol 14: R36. 23618408 10.1186/gb-2013-14-4-r36 PMC4053844

[31] Trapnell C, Williams BA, Pertea G, Mortazavi A, Kwan G, et al (2010) Transcript assembly and quantification by RNA-Seq reveals unannotated transcripts and isoform switching during cell differentiation. Nat Biotechnol 28: 511–515. 20436464 10.1038/nbt.1621 PMC3146043

[32] Trapnell C, Hendrickson DG, Sauvageau M, Goff L, Rinn JL, et al (2013) Differential analysis of gene regulation at transcript resolution with RNA-seq. Nat Biotechnol 31: 46–53. 23222703 10.1038/nbt.2450 PMC3869392

[33] Xu X, Zhang Y, Williams J, Antoniou E, McCombie WR, et al (2013) Parallel comparison of Illumina RNA-Seq and Affymetrix microarray platforms on transcriptomic profiles generated from 5-aza-deoxy-cytidine treated HT-29 colon cancer cells and simulated datasets. BMC Bioinformatics 14 Suppl 9S1. 10.1186/1471-2105-14-S9-S1 PMC3697991 23902433

[34] Al-Shahrour F, Minguez P, Tarraga J, Medina I, Alloza E, et al (2007) FatiGO +: a functional profiling tool for genomic data. Integration of functional annotation, regulatory motifs and interaction data with microarray experiments. Nucleic Acids Res 35: W91–96. 17478504 10.1093/nar/gkm260 PMC1933151

[35] Medina I, Carbonell J, Pulido L, Madeira SC, Goetz S, et al (2010) Babelomics: an integrative platform for the analysis of transcriptomics, proteomics and genomic data with advanced functional profiling. Nucleic Acids Res 38: W210–213. 20478823 10.1093/nar/gkq388 PMC2896184

[36] Huang da W, Sherman BT, Lempicki RA (2009) Systematic and integrative analysis of large gene lists using DAVID bioinformatics resources. Nat Protoc 4: 44–57. 19131956 10.1038/nprot.2008.211

[37] Caputo E, Manco G, Mandrich L, Guardiola J (2000) A novel aspartyl proteinase from apocrine epithelia and breast tumors. J Biol Chem 275: 7935–7941. 10713110 10.1074/jbc.275.11.7935

[38] Chu PG, Weiss LM (2002) Keratin expression in human tissues and neoplasms. Histopathology 40: 403–439. 12010363 10.1046/j.1365-2559.2002.01387.x

[39] Croce MV, Isla-Larrain MT, Demichelis SO, Gori JR, Price MR, et al (2003) Tissue and serum MUC1 mucin detection in breast cancer patients. Breast Cancer Res Treat 81: 195–207. 14620915 10.1023/A:1026110417294

[40] Echeverria PC, Picard D (2010) Molecular chaperones, essential partners of steroid hormone receptors for activity and mobility. Biochim Biophys Acta 1803: 641–649. 20006655 10.1016/j.bbamcr.2009.11.012

[41] Fritzsche FR, Dahl E, Pahl S, Burkhardt M, Luo J, et al (2006) Prognostic relevance of AGR2 expression in breast cancer. Clin Cancer Res 12: 1728–1734. 16551856 10.1158/1078-0432.CCR-05-2057

[42] Schnelzer A, Prechtel D, Knaus U, Dehne K, Gerhard M, et al (2000) Rac1 in human breast cancer: overexpression, mutation analysis, and characterization of a new isoform, Rac1b. Oncogene 19: 3013–3020. 10871853 10.1038/sj.onc.1203621

[43] Garcia-Murillas I, Sharpe R, Pearson A, Campbell J, Natrajan R, et al. (2013) An siRNA screen identifies the GNAS locus as a driver in 20q amplified breast cancer. Oncogene. 10.1038/onc.2013.202 PMC3970970 23752180

[44] Eswaran J, Cyanam D, Mudvari P, Reddy SD, Pakala SB, et al (2012) Transcriptomic landscape of breast cancers through mRNA sequencing. Sci Rep 2: 264. 22355776 10.1038/srep00264 PMC3278922

[45] Groshong SD, Owen GI, Grimison B, Schauer IE, Todd MC, et al (1997) Biphasic regulation of breast cancer cell growth by progesterone: role of the cyclin-dependent kinase inhibitors, p21 and p27(Kip1). Mol Endocrinol 11: 1593–1607. 9328342 10.1210/mend.11.11.0006

[46] Lange CA, Richer JK, Horwitz KB (1999) Hypothesis: Progesterone primes breast cancer cells for cross-talk with proliferative or antiproliferative signals. Mol Endocrinol 13: 829–836. 10379882 10.1210/mend.13.6.0290

[47] Zheng R, Blobel GA (2010) GATA Transcription Factors and Cancer. Genes Cancer 1: 1178–1188. 21779441 10.1177/1947601911404223 PMC3092280

[48] Myatt SS, Lam EW (2007) The emerging roles of forkhead box (Fox) proteins in cancer. Nat Rev Cancer 7: 847–859. 17943136 10.1038/nrc2223

[49] Chen HZ, Tsai SY, Leone G (2009) Emerging roles of E2Fs in cancer: an exit from cell cycle control. Nat Rev Cancer 9: 785–797. 19851314 10.1038/nrc2696 PMC3616489

[50] Simmen RC, Pabona JM, Velarde MC, Simmons C, Rahal O, et al (2009) The emerging role of Kruppel-like factors in endocrine-responsive cancers of female reproductive tissues. J Endocrinol 204: 223–231. 19833720 10.1677/JOE-09-0329 PMC2971688

[51] Kato S, Pinto M, Carvajal A, Espinoza N, Monso C, et al (2005) Progesterone increases tissue factor gene expression, procoagulant activity, and invasion in the breast cancer cell line ZR-75-1. J Clin Endocrinol Metab 90: 1181–1188. 15562024 10.1210/jc.2004-0857

[52] Fu XD, Giretti MS, Baldacci C, Garibaldi S, Flamini M, et al (2008) Extra-nuclear signaling of progesterone receptor to breast cancer cell movement and invasion through the actin cytoskeleton. PLoS One 3: e2790. 18665217 10.1371/journal.pone.0002790 PMC2464736

[53] Liang Y, Brekken RA, Hyder SM (2006) Vascular endothelial growth factor induces proliferation of breast cancer cells and inhibits the anti-proliferative activity of anti-hormones. Endocr Relat Cancer 13: 905–919. 16954439 10.1677/erc.1.01221

[54] Yee KO, Connolly CM, Duquette M, Kazerounian S, Washington R, et al (2009) The effect of thrombospondin-1 on breast cancer metastasis. Breast Cancer Res Treat 114: 85–96. 18409060 10.1007/s10549-008-9992-6 PMC2631620

[55] Wu J, Richer J, Horwitz KB, Hyder SM (2004) Progestin-dependent induction of vascular endothelial growth factor in human breast cancer cells: preferential regulation by progesterone receptor B. Cancer Res. 64: 2238–2244. 10.1158/0008-5472.can-03-3044 15026368

[56] Hyder SM, Murthy L, Stancel GM (1998) Progestin regulation of vascular endothelial growth factor in human breast cancer cells. Cancer Res 58: 392–395. 9458078

[57] Hyder SM, Chiappetta C, Stancel GM (2001) Pharmacological and endogenous progestins induce vascular endothelial growth factor expression in human breast cancer cells. Int J Cancer 92: 469–473. 11304678 10.1002/ijc.1236

[58] Hyder SM, Liang Y, Wu J, Welbern V (2009) Regulation of thrombospondin-1 by natural and synthetic progestins in human breast cancer cells. Endocr Relat Cancer 16: 809–817. 19570906 10.1677/ERC-08-0311

[59] Hubler TR, Scammell JG (2004) Intronic hormone response elements mediate regulation of FKBP5 by progestins and glucocorticoids. Cell Stress Chaperones 9: 243–252. 15544162 10.1379/CSC-32R.1 PMC1065283

[60] Shilatifard A (2012) The COMPASS family of histone H3K4 methylases: mechanisms of regulation in development and disease pathogenesis. Annu Rev Biochem 81: 65–95. 22663077 10.1146/annurev-biochem-051710-134100 PMC4010150

[61] Wertheimer E, Gutierrez-Uzquiza A, Rosemblit C, Lopez-Haber C, Sosa MS, et al (2012) Rac signaling in breast cancer: a tale of GEFs and GAPs. Cell Signal 24: 353–362. 21893191 10.1016/j.cellsig.2011.08.011 PMC3312797

[62] Fu XD, Goglia L, Sanchez AM, Flamini M, Giretti MS, et al (2010) Progesterone receptor enhances breast cancer cell motility and invasion via extranuclear activation of focal adhesion kinase. Endocr Relat Cancer 17: 431–443. 20233709 10.1677/ERC-09-0258

[63] Lain AR, Creighton CJ, Conneely OM (2013) Research resource: progesterone receptor targetome underlying mammary gland branching morphogenesis. Mol Endocrinol 27: 1743–1761. 23979845 10.1210/me.2013-1144 PMC3787126

[64] Pruitt KD, Tatusova T, Brown GR, Maglott DR (2012) NCBI Reference Sequences (RefSeq): current status, new features and genome annotation policy. Nucleic Acids Res 40: D130–135. 22121212 10.1093/nar/gkr1079 PMC3245008

[65] Wang JC, Derynck MK, Nonaka DF, Khodabakhsh DB, Haqq C, et al (2004) Chromatin immunoprecipitation (ChIP) scanning identifies primary glucocorticoid receptor target genes. Proc Natl Acad Sci U S A 101: 15603–15608. 15501915 10.1073/pnas.0407008101 PMC524211

[66] Carr HS, Zuo Y, Oh W, Frost JA (2013) Regulation of focal adhesion kinase activation, breast cancer cell motility, and amoeboid invasion by the RhoA guanine nucleotide exchange factor Net1. Mol Cell Biol 33: 2773–2786. 23689132 10.1128/MCB.00175-13 PMC3700125

[67] Papadimitriou E, Vasilaki E, Vorvis C, Iliopoulos D, Moustakas A, et al (2012) Differential regulation of the two RhoA-specific GEF isoforms Net1/Net1A by TGF-beta and miR-24: role in epithelial-to-mesenchymal transition. Oncogene 31: 2862–2875. 21986943 10.1038/onc.2011.457

[68] Kakinuma N, Zhu Y, Wang Y, Roy BC, Kiyama R (2009) Kank proteins: structure, functions and diseases. Cell Mol Life Sci 66: 2651–2659. 19554261 10.1007/s00018-009-0038-y PMC11115667

[69] Wang Y, Onishi Y, Kakinuma N, Roy BC, Aoyagi T, et al (2005) Alternative splicing of the human Kank gene produces two types of Kank protein. Biochem Biophys Res Commun 330: 1247–1253. 15823577 10.1016/j.bbrc.2005.03.106

[70] Soundararajan R, Wang J, Melters D, Pearce D (2007) Differential activities of glucocorticoid-induced leucine zipper protein isoforms. J Biol Chem 282: 36303–36313. 17956870 10.1074/jbc.M707287200

[71] Redjimi N, Gaudin F, Touboul C, Emilie D, Pallardy M, et al (2009) Identification of glucocorticoid-induced leucine zipper as a key regulator of tumor cell proliferation in epithelial ovarian cancer. Mol Cancer 8: 83. 19814803 10.1186/1476-4598-8-83 PMC2763858

[72] Meyer CA, Tang Q, Liu XS (2012) Minireview: applications of next-generation sequencing on studies of nuclear receptor regulation and function. Mol Endocrinol 26: 1651–1659. 22930692 10.1210/me.2012-1150 PMC3458226

[73] Hah N, Kraus WL (2013) Hormone-regulated transcriptomes: lessons learned from estrogen signaling pathways in breast cancer cells. Mol Cell Endocrinol 382: 652–664. 23810978 10.1016/j.mce.2013.06.021 PMC3844033

[74] Ballare C, Castellano G, Gaveglia L, Althammer S, Gonzalez-Vallinas J, et al (2013) Nucleosome-driven transcription factor binding and gene regulation. Mol Cell 49: 67–79. 23177737 10.1016/j.molcel.2012.10.019

[75] Clarke CL, Graham JD (2012) Non-overlapping progesterone receptor cistromes contribute to cell-specific transcriptional outcomes. PLoS One 7: e35859. 22545144 10.1371/journal.pone.0035859 PMC3335806

[76] Yin P, Roqueiro D, Huang L, Owen JK, Xie A, et al (2012) Genome-wide progesterone receptor binding: cell type-specific and shared mechanisms in T47D breast cancer cells and primary leiomyoma cells. PLoS One 7: e29021. 22272226 10.1371/journal.pone.0029021 PMC3260146

