# Supplementary figures and images for: Deep Sequencing Reveals New Aspects of Progesterone Receptor Signaling in Breast Cancer Cells
Source: PLoS One. 2014 Jun 4;9(6):e98404. doi: 10.1371/journal.pone.0098404 (PMC4045674; doi:10.1371/journal.pone.0098404)

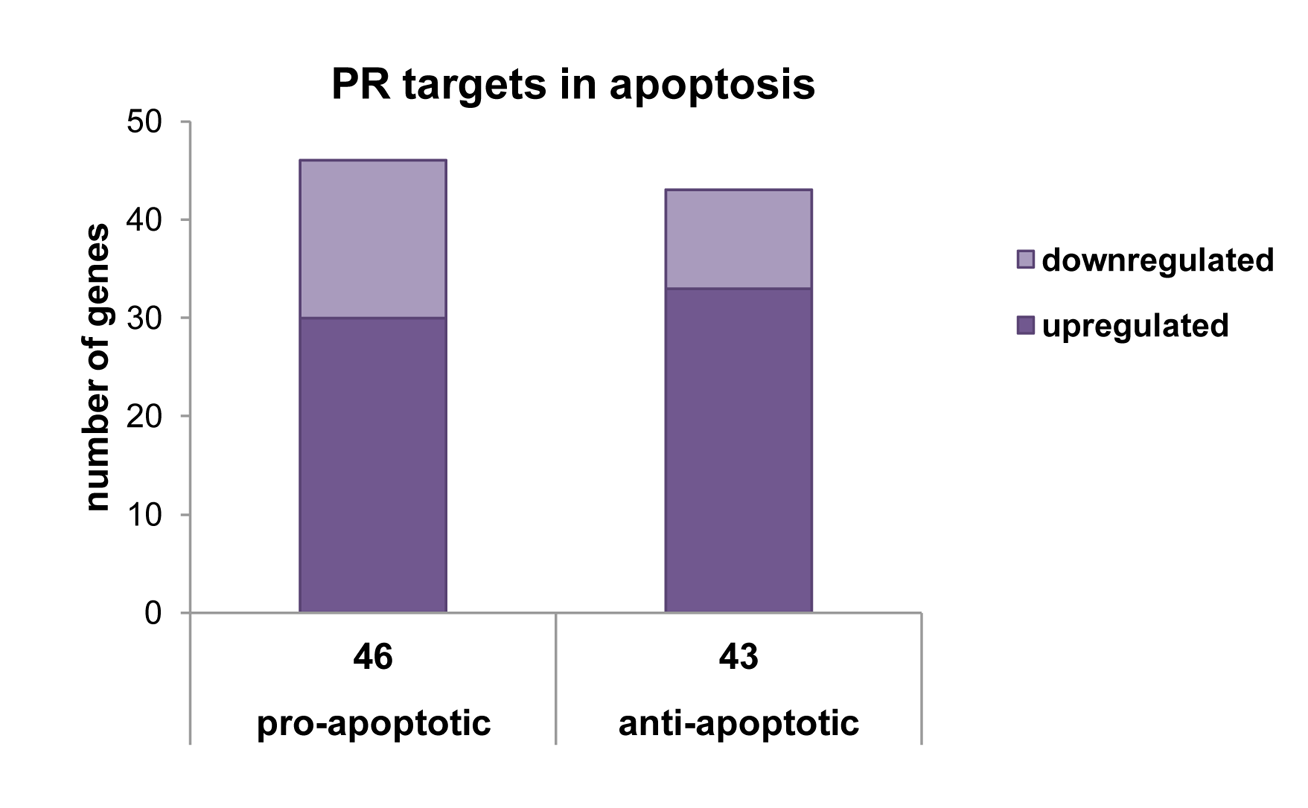

Supplement: Figure S1 — PR-regulated genes involved in cell death/apoptosis. GO annotation and literature search led to the functional categorization of genes involved in cell death as pro-apoptotic or anti-apoptotic. A few genes were denoted as both pro- and anti- apoptotic and were counted in both groups. (TIF) [file pone.0098404.s001.tif]

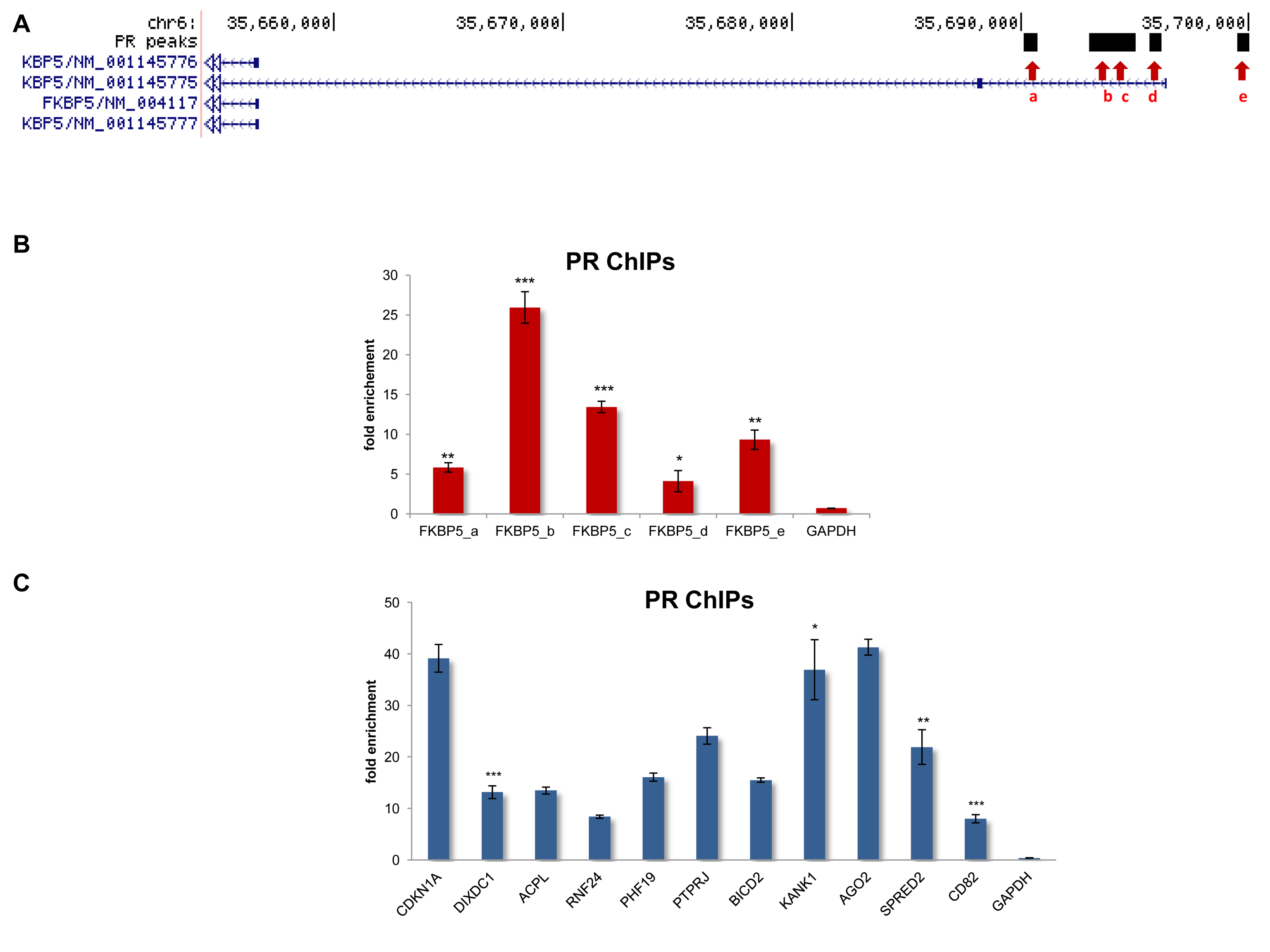

Supplement: Figure S2 — ChIP-sequencing experiments for the PR identify receptor binding sites in T47D cells. (A) Cells treated with R5020 for 1 hr were used for ChIP experiments with an antibody against the PR. Immunoprecipitated DNA was used in sequencing experiments and representative data are shown here. Four PR binding sites (depicted by black blocks) were found in distal enhancer elements of FKBP5 transcript variant 1 (NM_004117), which is the PR-regulated transcript. (B) ChIP-qPCR experiments for the PR were performed in progestin- and vehicle- treated cells. The primers used amplified part of some of the PR binding sites (depicted by red arrows and labeled a-e) identified in the FKBP5 locus by ChIP-seq. Error bars indicate the SEM. (single asterisk indicates p-value<0.05, double asterisk p-value<0.005 and triple asterisk p-value<0.005). (C) As in (B), but primers used amplified part of the PR binding sites associated with the genes shown. A known PR binding site in the promoter of CDKN1A is used as a positive control and the promoter of GAPDH as a negative one (p-value<0.001, single asterisk indicates p-value<0.05, double asterisk p-value<0.01 and triple asterisk p-value<0.005). (TIF) [file pone.0098404.s002.tif]
